# The Use of Virtual Reality Alone Does Not Promote Training Performance (but Sense of Presence Does)

**DOI:** 10.3389/fpsyg.2020.01743

**Published:** 2020-07-17

**Authors:** Simone Grassini, Karin Laumann, Martin Rasmussen Skogstad

**Affiliations:** ^1^ Department of Psychology, Norwegian University of Science and Technology, Trondheim, Norway; ^2^ NTNU Social Research, Studio Apertura, Trondheim, Norway

**Keywords:** head-mounted displays, virtual reality, presence, performance, training, human factors

## Abstract

Virtual reality (VR) offers novel ways to develop skills and learning. This technology can be used to enhance the way we educate and train professionals by possibly being more effective, cost-efficient, and reducing training-related risks. However, the potential benefits from virtual training assume that the trained skills can be transferred to the real world. Nevertheless, in the current published scientific literature, there is limited empirical evidence that links VR use to better learning. The present investigation aimed to explore the use of VR as a tool for training procedural skills and compare this modality with traditional instruction methods. To investigate skill development using the two forms of training, participants were randomly divided into two groups. The first group received training through an instructional video, while the second group trained in VR. After the training session, the participants performed the trained task in a real setting, and task performance was measured. Subsequently, the user’s experienced sense of presence and simulator sickness (SS) was measured with self-report questionnaires. There were no significant differences between groups for any of the performance measures. There was no gender effect on performance. Importantly, the results of the present study indicate that a high sense of presence during the VR simulation might contribute to increased skill learning. These findings can be used as a starting point that could be of value when further exploring VR as a tool for skill development.

## Introduction

The field of immersive visual technology, and specifically virtual reality (VR), provides several novel approaches for learning and skill development. Along with these newfound approaches, VR might improve how we teach and train professionals by possibly being more effective, eliminating numerous barriers, and reducing the cost compared to traditional training methods. Nevertheless, the potential benefits of VR rest on the assumption that the trained ability can be effectively transferred to real-life performances, and that the VR training outcomes are somehow similar or better compared to regular education and training techniques.

Limited empirical scientific work has examined the advantages of utilizing immersive VR for learning and skill training. Furthermore, the current research has inconsistent outcomes (e.g., Makowski et al., 2017; Makransky et al., 2019). Nevertheless, VR has been used for training purposes with the hope that with the increased immersive quality of the technology—and therefore the elicited subjective sense of presence—may have a positive effect on training (Slater and Wilbur, 1997; Bodekaer, 2016). Thus, it is important to verify the possible advantages of using VR technology in skill training, and these possible benefits should be contemplated in the framework of economical and practical considerations before the technology will be ready to be implemented for industrial and educational purposes.

The scientific literature is still at the beginning of the investigation of the training effect of immersive VR, and whether VR training may provide objective advantages in task performance should be further investigated. However, it is worth to mention that a number of studies support the idea that VR offers advantages compared to traditional training methods (see e.g., Cryer et al., 2019), and that VR training can increase performance (Alhalabi, 2016; Passig et al., 2016; Webster, 2016), even though these findings have been recently challenged (Makransky et al., 2019, 2020).

Moreover, previous studies have primarily tested the ability to remember and report procedures learned in VR, rather than objectively testing if the learned information was useful to improve task performance in a real setting (e.g., Dubovi et al., 2017; Makowski et al., 2017; Jung and Ahn, 2018). The sense of presence during a VR experience has been linked to better learning, but on the other hand, also to a higher cognitive load for the users (that may reduce the allocation of cognitive resources to learning, see Makransky et al., 2019).

Understanding the level of training can be achievable using a “surrogate” of the real task (e.g., a video or a VR simulation) and understanding which administration method of the training section is crucially important for applied conditions. In work environments, for example, employers can often not train with the “real” task (e.g., for cost or safety reasons), and therefore they need to be trained beforehand with a “surrogate” task. VR has in recent years found space in work and organizational environments (see e.g., Grassini and Laumann, 2020b; Saghafian et al., 2020).

The research questions we aim to answer in the present investigations are: (1) does VR-based learning help procedural learning compared to a two-dimensional (2D) screen-based video-tutorial? (2) Does gender affect learning outcomes? (3) What is the relationship between experienced sense of presence, simulator sickness (SS), and the training outcomes in the VR condition?

### VR: Immersion and Presence

VR enables the presentation of artificial settings that represent possible scenarios in a realistic and immersive way. VR offers a continuous spatial and temporal experience that ideally serves the purpose of enhancing the user’s subjective feeling of “presence” (Metzinger, 2018). With respect to the effect of the technology on users, there remains confusion regarding the terms “immersion” and “presence.” In a milestone work, Slater (2003) suggested that the word “immersion” often refers to the objective technical attributes of the device being used. The term thereby refers to the degree to which the technical system manages to deliver an experience as close to the “real-life experience” as possible, including its sensory modalities. According to Slater (2003), immersion is a concept connected to—but not equivalent to—presence. However, some scholars have used the two terms interchangeably. Presence refers to the human, subjective perception of the virtual experience, “a human reaction to immersion” (Slater, 2003, p. 2). Presence (sometimes referred also as “sense of presence”) is commonly described as the sensation of “being there.” Moreover, presence is a subjective psychological construct, and its measurement and investigations have been challenging (see Grassini and Laumann, 2020a). This fact contrasts with immersion, which is—or should theoretically be—a relatively more objective and unbiased measure.

### VR Technology in Training

The high level of immersion provided by modern head mounted display (HMD) VR, and therefore its ability to stimulate the sense of present of the user, could improve the way skills are trained in several fields, including education and business. First, VR technology might prove to be more cost-effective compared to standard training methods. VR can reduce the demand for human instructors, enable training of skills and procedures regardless of the physical location of the trainee, reduce the demand of travels, and reduce the needs to produce training materials. Moreover, reduced travel and equipment manufacturing could help improve the environmental sustainability of training processes. The use of VR technology would prove to be particularly beneficial in these applications where training tools are limited and/or expensive, in those environments where competent instructors are lacking, and in those situations where training involves dangerous materials (Bal, 2012). For instance, the use of VR for training has been adopted for activities regarding safety and hazards in chemical industrial plants (Bell and Fogler, 2000). Training in VR has also been employed in the energy and oil industry (Brasil et al., 2011). Implementing VR could also be beneficial for training health care professionals, including doctors and nurses. In the medical field, the use of VR may have ethical implications, ultimately reducing the need for *in vivo* animal research and cadavers in training (Logishetty et al., 2019). Finally, the use of VR could enable easier and more accurate data collection on training progress by making training processes easier and providing the ability to give better feedbacks to the trainees. Training-related data could be used to track personal progress while also informing institutions and stakeholders of general training parameters that could be used to improve the training process itself.

From a theoretical perspective, it has been proposed that the immersive VR should foster learning (Slater and Wilbur, 1997; Salzman et al., 1999; Lee et al., 2010). According to those theories, the increased level of immersion achievable by VR technology may promote engagement and motivation, and consequently, better cognitive processing of the learning material.

Several studies have been conducted to evaluate the influence of VR on training outcomes, and empirical reports have showed heterogeneous results (Dubovi et al., 2017; Gonzalez-Franco et al., 2017; Makowski et al., 2017; Jung and Ahn, 2018; Makransky et al., 2019). Logishetty et al. (2019) found that surgeons trained using VR were more accurate in performing a total hip arthroplasty procedure compared to those that had undertaken the standard training. The surgeons trained with VR were able to complete 33% more key steps and they performed the surgery 18% faster compared to the trainees who did not use VR. A further investigation explored the difference in procedural learning of medication administration procedures between a VR training program and standard lecture-based training, finding that the VR training facilitated conceptual-procedural learning (Dubovi et al., 2017). Other studies, however, have found a negative or no correlation between the use of VR and learning (Vélaz et al., 2014; Makransky et al., 2019). Although participants reported an increased sense of presence when trained in an immersive VR environment, this phenomenon resulted in a poorer learning experience compared to training with less immersive technologies (Makransky et al., 2019). The latter study employed psychophysiological variables to try to understand cognitive processes during the learning experience. The electroencephalogram (EEG) recordings showed increased cognitive load in the VR environment. Therefore, Makransky et al. (2019) suggested that immersive VR does not improve learning due to the higher cognitive demands of the medium compared to traditional learning. On a similar note, Bailey et al. (2012) indicated that humans may have limited cognitive resources to allocate to the relevant tasks at hand when contemporarily navigating and perceiving the virtual world. Contrary to Bailey et al. (2012) and Makransky et al. (2019) found a negative correlation between the reported level of presence and the ability to remember information after learning it in a virtual environment. Based on these results, Bailey et al. (2012) concluded that a highly vivid and sensory-heavy experience presented in the virtual environment might increase the sense of presence while simultaneously depleting the available cognitive resources, a phenomenon that would inhibit the ability for memory encoding.

### Gender Differences and the Use of VR

Recently, some investigations have argued that HMDs may have negative effects specifically for female users (e.g., Munafo et al., 2017). However, these data have often only focused on negative symptoms from simulator sickness (SS) and were not derived from investigating other factors, such as performance and possible learning differences between males and females in VR (see Grassini and Laumann, 2020c, for a recent review). Few articles have reported gender differences in performance connected to the use of VR, and results have often shown no differences (Cárdenas-Delgado et al., 2017; Juan et al., 2018; Khashe et al., 2018; Roettl and Terlutter, 2018). However, a minority of studies have shown that females may perform better in tasks related to VR (Allen et al., 2016; Liang et al., 2019). The higher sense of presence sometimes reported by female compared to male participants (see e.g., Gamito et al., 2008) may be associated with the increase performance in female participants, as performance and sense of presence have been found to be associated in learning activities (Yang et al., 2016).

## Materials And Methods

The present study used a between-subject design. Participants were randomly divided into two groups, in which one participated in a training session by watching an instructional video on a 2D LCD screen, while the other group participated in a training section using HMD VR.

### Study Sample

Participants were recruited among volunteers of the student population at the Norwegian University of Science and Technology. The study, as well as the informed consent, was approved by the Norwegian Centre for Research Data (NSD) prior to the start of the experiment. The study was conducted according to the guidelines of the Declaration of Helsinki.

A total of 30 participants (12 men and 18 women) participated in the present study. The participants were on average 24.8 years old (*SD* = 3.01), however only data from 29 participants were used for the analysis of performance statistics, and data from 27 were used in the analysis involving questionnaire scores (see “Results”). All the participants self-reported to be right-handed with no intake of psychotropic drugs, history of psychiatric/psychological illness, photosensitivity, or epilepsy seizures. The sample size was chosen to be in line with the ones reported by recently published papers that have investigated the effect of VR on training (An et al., 2018; Bracq et al., 2019; Liang et al., 2019).

### Training, Task, and Experimental Procedure

The task in which the participants trained comprised building a small model of an airplane, using building blocks of various colors. Participants were assigned to the 2D- or VR-based training according to their order of arrival in the lab; each group consisted of 15 participants. After their arrival, the participants were given information on the experiment and asked to read and sign the informed consent.

In the 2D condition, the participants passively watched an instructional video. In the VR condition, the participants were asked to perform the building task following some instructional images while wearing a HMD and operating in the environment using joypads. At the beginning of the VR condition, the participants were asked to sit on a chair positioned in the middle of the lab room, away from possible obstacles. The VR headset was then fitted on their head, and they were instructed on how to modify the inter-pupillary distance (IPD), to adapt it to their own eye distance. The participants were then instructed on the controller functions (e.g., how to move the pieces on the virtual table and which joypad buttons were to be used). It was clarified to the participant that the experiment would last at maximum of 10 min, or until the task was fully completed (the item was built correctly according to instructions displayed in the virtual environment). The participants were further helped in case they asked the experimenters, and they were solicitate to ask for help in case they were unable to perform some movements or were experiencing problems in operating in the virtual scene. Furthermore, the experimenter reminds the participants that they could stop the experiment at any moment. None of the participants decided to prematurely terminate the experiment.

Both participant groups were then asked to perform the trained task on a real model, identical to the one shown in the 2D and VR training. Performance metrics were recorded to assess training outcomes. The 2D video was approximately 2 min, while the VR group was instructed to interact in the VR environment up to a maximum 10 min (to allow the participants to get used to the VR and accommodate the novel environment), or until the building task was fully and correctly completed. Unfortunately, it is not possible for us to report in the present article images of the experimental training conditions or the task, as it would violate the copyright of the utilized materials. We encourage authors willing to replicate the present study to contact the correspondent author of the present article.

After the training was completed, the participants were asked to perform the building task with a real model placed on a table. A photo of the final product was also on the table. After the task was terminated, the participants who performed the VR training session were asked to complete the presence questionnaire (PQ) and the simulation sickness questionnaire (SSQ). Please note that the questionnaires were given only to the VR-training participants and not to the participants of the 2D condition. The decision to have such “inequality” in the two experimental conditions was taken after an earlier pilot of the study showed that many participants were confused on how to interpret the question of the PQ (e.g., “how completely were your sense engaged” and “how much were you able to control events”) to a simple 2D video. Furthermore, some of the pilot participants were confused on the degree of discomfort that the 2D video should have provoked (according with the items of the SSQ), as none of them generally experience discomfort on watching a 2D video. However, we can assume that the degree of presence would be generally lower for 2D video compared to VR, and that 2D video would promote a lower degree of SS compared to VR (however SS was reported in some experiment, also for passive watching of desktop video displays, see e.g., Dong et al., 2011).

### Equipment

The instructional video used in the 2D condition was filmed with a 48-megapixel (MP) camera; it was later edited to enhance the color quality of the recording and contrast. The video was filmed in a first-person view; it showed a table where both hands of the instructor were visible, and the building blocks were laid down on a wooden table, maintaining the identical order as in the VR condition. The plane was assembled in steps, with pauses between each step and close-ups of some of the bricks being used.

A Windows-based stationary computer was used for the 2D training session. The system featured a 15-inch LCD display with a resolution of 1,920 × 1,080 pixels. The video was played using YouTube Player, with 720 p video quality. The participants were seated approximately 60 cm away from the computer when the video was shown. The video did not feature sound. Two images, one showing the final product to be built and one an instruction sequence list, were shown in the video.

The VR equipment used for this study was an HTC-Vive Pro with a 2,880 × 1,600 pixel resolution, two position sensors, and two controllers (standard bundle). The participants used an application specifically developed for the present study, featuring a warehouse background and a table with the identical building block pieces from the building blocks set. The same two instruction images that appeared in the 2D version of the training were shown during the VR presentation.

During the real-life task (experimental task), the assembly procedure was filmed to allow offline analysis and estimation of the performance metrics of the participants. These videos only contained the hands of the participants and an overview of the table where the building blocks were placed.

### Questionnaire Instruments

Questionnaires were used to evaluate the subjective experience of the participants during the test. The subjects were asked to complete the questionnaires immediately at the end of the experimental task, in the order described below.

#### Presence Questionnaire

The PQ (Witmer et al., 2005) was used; specifically the version revised by UQO Cyberpsychology Lab (2004). The PQ used in this experiment consisted of 19 items divided into five domains (“realism,” “possibility to act,” “quality of interface,” “possibility to examine,” and “self-evaluation of performance”). Each question uses a seven-point Likert-type scale. Therefore, each participant could achieve a total maximum score of 133. The PQ is one of the most commonly used questionnaires in VR research (Hein et al., 2018). The version of the PQ used in the present study is considered to have good internal validity (Cronbach’s *α* = 0.84; UQO Cyberpsychology Lab, 2004), and the PQ has shown to be robust and reliable to analyze the sense of presence (Witmer et al., 2005).

#### Simulation Sickness Questionnaire

The SSQ was used to assess to what degree participants experienced motion sickness, or SS, while training. The SSQ has been widely used to evaluate and explore any significant effects on SS (e.g., Lin et al., 2002). Each question (16 in total) is linked to a symptom of motion sickness—nausea, oculomotor, and disorientation—and should be answered according to the severity of each symptom ranging from “none” to “severe.” The SSQ is considered to provide good indications of overall SS severity and reasonable powerful subscale scores for diagnostic purposes (Kennedy et al., 1993). In the present study, the total score of SSQ was used as an overall index of SS symptom severity.

### Performance Measures

The measures used for estimating the participants’ performance were product quality, errors made during assembly, and speed of assembly. Product quality was defined as whether the building block plane was correctly assembled, so that the product was correct according to the instruction given during the training phase. The product quality was assessed as a binary variable based on the correctness of the final product (complete/incomplete).

All the performance measures were evaluated analyzing the video recordings of the experimental sessions. One of the experimenters was in charge of coding the performance measures. This person received the recording in batches and was unaware of the training condition that the subject was coding until all the coding was finalized.

The number of errors was scored as the number of mistakes made (a piece not placed correctly). An error during assembly was defined as a participant misplacing a block and then picking another block. If the model blocks containing errors were accidentally disassembled (e.g., by dropping the model), reassembling the model to its previous form (including errors) did not contribute to the error count. However, if the model was disassembled intentionally, replicated errors would be counted as newly performed errors, even if they were identical to previous ones. Misplacements were judged based on the function of a building block and its relation to neighboring ones. However, even though the misplacement of one block is inevitably linked to the misplacement of at least one other block, this phenomenon was counted as only one error.

Completion speed was measured in seconds from when the participants first picked up a piece of building block during the test phase until they indicated they were done. Three questionnaires were used to assess motion sickness, presence, and the quality of the VR software.

### Data Analysis and Statistics

The participants’ performance metrics for continuous variables (errors and speed) were compared between the two experimental conditions (2D and VR training) using independent samples *t*-tests. A chi-square test was used to compare the two experimental conditions for the performance metric “product quality” (complete/incomplete). The same type of analyses was used to assess the differences in PQ and SS between the experimental groups.

In the following analyses, independent samples *t*-tests were used to assess possible performance differences as well as difference in experienced PQ and SS using participant’s gender as grouping variable. With regard to using the *t*-test, Levene’s test showed that the equality of variance was violated. Hence, we report *t* and *p* values corrected for violation of equality of variance.

Two-tailed correlation tests were performed to understand the relationships between the explorer variables. Pearson correlations were performed for continuous variables (speed and errors). Two-tailed correlation analyses (Spearman’s Rho) were computed to assess a possible association when non-continuous variables were included (product quality and sense of presence).

## Results

One participant (from the VR training group) was excluded because he or she performed significantly worse than the others in the number of errors metric (over two SDs from the mean). Two participants in the VR group and one participant in the 2D group did not complete the PQ and SSQ inventories. These three participants were therefore excluded from data analysis involving these questionnaires. An independent samples *t*-test was used to assess possible significant differences in the age distribution across the two experimental groups; there was no difference (*p* = 0.953). Although the participants were instructed to terminate the VR experience in the case of a high level of discomfort during the simulation, none of the participants decided to prematurely terminate the experiment.

### Descriptive Statistics

The participants in the 2D instructional video training group (*N* = 15, seven males and eight females) were on average 24.93 years old (*SD* = 3.35). Forty-seven percent of them correctly built the real model of the airplane in the experimental task. The average task completion time was 250.80 s (*SD* = 131.86), and the average number of errors performed during the building task was of 6.13 (*SD* = 4.93).

The participants in the VR training group (*N* = 14, five males and nine females) had an average age of 24.87 years old (*SD* = 2.70). Thirty-six percent of them correctly built the real model of the airplane in the experimental task. The average task completion time was 316.25 s (*SD* = 194.91), and the average number of errors performed during the building task was 6.14 (*SD* = 5.30). They scored an average of 4.49 (*SD* = 1.58) in the PQ and 20.57 (*SD* = 17.50) in the SSQ.

For the VR condition, the PQ mean score is in line with the score of 4.65 reported in Kober and Neuper (2013). However, please note that those studies did not use an HMD. The SSQ results in the present study were significantly lower compared to those reported in previous studies compared to other recent studies using HMDs (e.g., Kim et al., 2018, which reports scores from 28.67 to 42.66; Somrak et al., 2019, which reports from 45.95 to 56.41 for different HMD models).

### Performance Metrics

**Figure 1:**
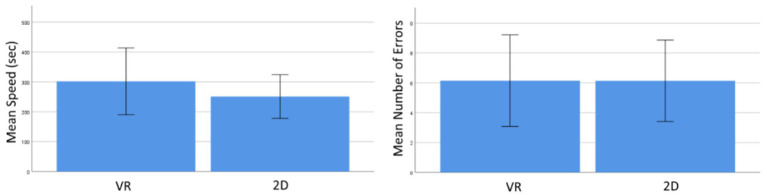
Mean speed (left) and number of errors (right) for subjects participating in the virtual reality (VR) or two-dimensional (2D) training session. The error bars represent *SD*.

There was no significant difference between the two training conditions for errors made during the assembly task [Figure 1, left graph, *t*(27) = 0.005, *p* = 0.996]. The participants trained in VR spent more time completing the real task, but this effect was not significant [Figure 1, right graph, *t*(27) = 0.84, *p* = 0.411]. The proportion of participants who correctly assembled the item did not differ between groups [*χ* ^2^ (1, *N* = 29) = 0.36, *p* = 0.55]. Not surprisingly, due to the different media in which the training was presented, participants in the VR group reported an overall higher sense of presence during the training (PQ score) compared to the 2D group [*t*(24) = 2.92, *p* = 0.008].

### Gender Differences in Performance and Sense of Presence

**Figure 2:**
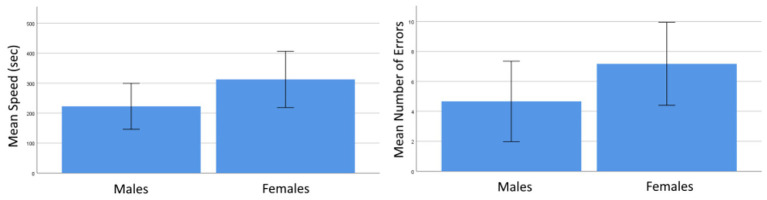
Mean speed (left) and number of errors (right) for males and females. The error bars represent *SD*.

When the 2D and VR conditions were considered together, *t*-tests revealed that there were no differences between the genders for completion speed [*t*(27) = −1.49, *p* = 0.149] or number of errors [*t*(27) = −1.345, *p* = 0.190]. The proportion of participants who correctly assembled the item did not differ by gender [*χ* ^2^ (1, *N* = 29) = 0.001, *p* = 0.979]. Figure 2 shows an overview of performance means by gender. Male and female participants in the VR training group did not differ with regard to the reported sense of presence [*t*(10) = 1.317, *p* = 0.217] or reported SS [*t*(10) = −0.505, *p* = 0.624].

To study possible gender differences in the performance outcomes from the two different training conditions, *t*-tests were computed separately for the 2D and VR training, using gender as a grouping variable. For the 2D training group, there were no gender differences with regard to task completion speed [*t*(13) = −0.612, *p* = 0.551] or number of errors [*t*(13) = −1.047, *p* = 0.309]. There was also no difference for product quality [*χ* ^2^ (1, *N* = 15) = 0.077, *p* = 0.782].

**Figure 3:**
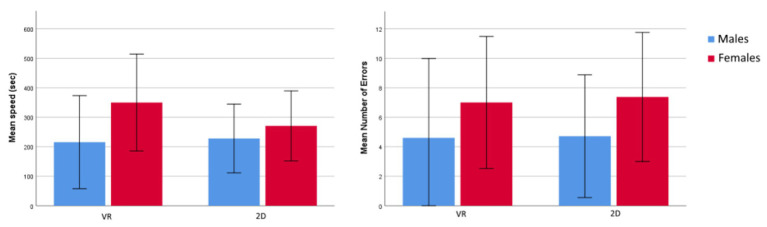
Mean speed (left) and number of errors (right) for males and females, divided by the type of training received prior to the building task. The error bars represent *SD*.

For the VR training group, there was no difference between the genders for completion speed [*t*(12) = −1.27, *p* = 0.228] or number of errors [*t*(12) = −0.800, *p* = 0.439]. Further, there was no difference for product quality [*χ* ^2^ (1, *N* = 14) = 0.062, *p* = 0.803]. Figure 3 shows an overview of performance means between gender, divided by training type. Male and female participants did not differ in the subjective experience of sense of presence in VR [*t*(10) = 1.32, *p* = 0.217] or SS [*t*(10) = −0.505, *p* = 0.624].

### Correlation Analyses

**Figure 4:**
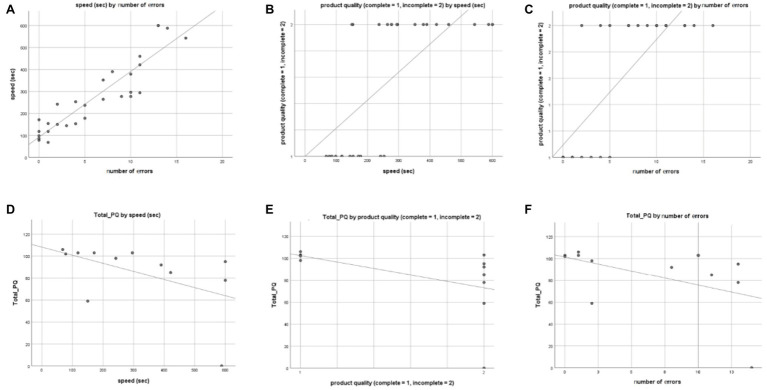
Scatter plots of statistically significant correlations. Task completion speed x number of errors (A), product quality x task completion speed (B), product quality x number of errors (C), total PQ score x task completion speed (D), total PQ score x product quality (E), and total PQ score x number of errors (F). Linear relationships are indicated.

A parametric correlation analysis (considering both 2D and VR training groups) revealed that completion time and number of errors were positively correlated (*Rho* = 0.917, *p* < 0.001, Figure 4A). This finding indicates that participants who made more errors were also slower at assembly. Non-parametric correlations showed that product quality positively correlated with task completion speed (*Rho* = 0.753, *p* < 0.001, Figure 4B) and number of errors (*Rho* = 0.811, *p* < 0.001, Figure 4C).

For the VR condition, sense of presence was highly associated with all the performance metrics. Participants who experienced a higher sense of presence during the VR training performed significantly better in the block building task (*Rho* = −0.632, *p* = 0.028, for completion speed – Figure 4D; *Rho* = −0.715, *p* = 0.009, for product quality; *Rho* = −0.681 – Figure 4E, *p* = 0.015, for number of errors – Figure 4F). The SSQ score did not correlate with performance metrics (*ps* > 0.523) or sense of presence (*p* = 0.545).

## Discussion

The present investigation aimed to compare two tools: learning by watching a task being performed in an instruction-like video on a 2D screen or learning by performing the task first-hand in interactive and immersive VR. The learning and performance task involved building; specifically, the participants were instructed on how to build a small airplane model using plastic building blocks. The focus of the experiment was to assess the performance of the participants after they had experienced one of the two different trainings. Task performance was measured using a real-life task right after one of the two training session types. The performance metrics chosen were (1) the speed of completion of the item, (2) number of errors made during building, and (3) quality of the finished product (complete/incomplete). The data showed that there were no significant differences in performance metrics between the 2D and VR groups. Male and female participants did not differ in their performance, regardless of the training modality. All the investigated metrics (completion, errors, and product quality) were associated with the reported sense of presence experienced in the VR training: participants who experienced a higher sense of presence performed better in the actual task.

According to Makransky et al. (2019), the use of VR technology is associated with an increased cognitive load that takes away cognitive resources from the learning experience. If such an effect is generalizable to our training environment, we would expect a learning advantage for the participants training using the 2D video compared to VR. In contrast, if the increased sense of presence in VR improves engagement in learning activities, as suggested by Salzman et al. (1999) and Lee et al. (2010), there should be an advantage for the group of people trained using VR.

In the present investigation, there were no significant differences in task performance (task speed, errors, and product quality) between the 2D video and VR training groups. Furthermore, gender did not affect performance or the subjective perception of the VR environment (elicited sense of presence and SS). However, consistent with the ideas proposed by Salzman et al. (1999) and Lee et al. (2010), there was an association between sense of presence and performance metrics, with participants who experienced a higher sense of presence scoring better in all three performance metrics. Such a finding suggests that increased presence does contribute to augment skill learning, contrary to the hypothesis proposed by Makransky et al. (2019). That study linked the use of VR to increased cognitive load and used psychophysiological metrics (EEG spectral decomposition) as an index of workload.

The present study did not use a direct or indirect measure for evaluating cognitive load; however, future studies may use psychophysiological metrics to assess the users’ cognitive load during the learning experience. This endeavor would add further insight on how VR can be successfully used as a learning tool. Specifically, electrophysiological brain correlates of workload (e.g., reduction in alpha and increase in theta waves during electroencephalography recording) have been often reported in the literature as an index of cognitive load (Gevins and Smith, 2003; Antonenko et al., 2010; Grassini et al., 2019).

SS experienced during the VR experience did not impact the participants’ performance. However, this phenomenon may be due to the overall low SS score reported for the VR simulation used in the present experiment compared to previous investigations (see Somrak et al., 2019). A low degree of experienced SS in our VR environment was indirectly confirmed by the fact that no participant decided to quit the experiment due to SS symptoms. It is logical to think that VR environments that promote a higher level of discomfort may have a greater effect on task performance.

Male and female participants did not show any difference in performance. There was also no gender difference in the level of experienced sense of presence either, findings that are contrary to previous studies that have demonstrated females experience a higher level of presence and, consequently, perform better (Allen et al., 2016; Liang et al., 2019). Nevertheless, our results confirmed previous findings were gender was not found to be associated with task performance differences (Cárdenas-Delgado et al., 2017; Juan et al., 2018; Khashe et al., 2018; Roettl and Terlutter, 2018).

The present study aimed to determine whether one learning method was better than the other. Intriguingly, the results indicated no significant difference between the two methods; consequently, the question about what method is best relies on context and weighing the pros and cons. Several studies have suggested that immersive VR could potentially improve the way skills are taught and practiced in education, industry, and health care (e.g., Slater and Wilbur, 1997; Salzman et al., 1999; Lee et al., 2010; Bal, 2012; Logishetty et al., 2019). This statement is based on different benefits that VR introduces in such environments. The results from the present study indicate that learning through doing in VR does not improve on the method of watching an instruction video, and thus the other potential benefits of VR need to be considered when contemplating VR as a learning tool. VR could make the need for instructors unnecessary, in addition to enabling training of procedures and skills regardless of place and position. In other words, VR enables one to bring the appropriate context and situation to a person wherever he or she may be. For example, people could be trained in different types of lab work without having them in the lab. While this point could also be made for an instructional video, the situation could be different for more complex tasks and environments. As mentioned above, VR could potentially benefit industries where training requires limited—or expensive—physical equipment and competent instructors, or in industries where training involves dangerous materials (Bal, 2012).

A VR environment allows one to move in space and interact with the objects presented and learn manual skills. The latest developments in VR (see the hand-tracking system recently released for Oculus Quest) may allow for more precise hand movements and, therefore, increase the benefits in learning those skills were fine-tuned movements are crucial.

### Limitations

It should be noted that the comparisons in this study were made with a fairly small sample size (but in line with previous similar studies, see An et al., 2018; Bracq et al., 2019; Liang et al., 2019). A larger sample size would increase the statistical power; for example, the inclusion of participants with a higher degree of SS may provide a better understanding on how discomfort during the VR simulation may affect task performance.

The two conditions (VR and 2D) were quite different as a mean of presentation of the stimuli. However, such limitation is unavoidable when a direct comparison wants to be made between very different means of visualization that uses different technologies.

A major limitation of the present investigation is that the practice sessions were quite different between the two experimental groups during the training session. In the 2D condition, participants were shown a 2-min video that presented a person assembling the model, but they did not have any control over the video streaming. The participants were unable to change the video’s speed; freeze the screen to double check what block was being used in the process, or repeat the video. These conditions are unrealistic, as in a real scenario for work training; the participants in the training sessions have the possibility to manipulate the simulation to maximize the gathering of relevant information.

By contrast, the VR group was given 10 min to complete a model; the participants could freely assemble and disassemble the model until they were content. Their trial session ended when they completed the model, but until that time, the participants could look at the instruction multiple times, to check the blocks and make sure they were using the right ones. The experimental design was chosen considering the possible limitations as earlier pilot of the study showed that participants in the VR-condition needed a significant amount of time to adapt to the use of the controllers and to adequately being able to navigate the virtual scene.

Future studies could consider allowing participants in a 2D condition more control, i.e., pausing the video, in order to make the conditions more similar. This approach would also improve the ecological validity of the results for real-life trainings.

The setup in this experiment was based on standardizing and limiting the number of times participants could build the model or see the model being built. This setup was chosen to avoid ceiling effects, which could occur if all participants had the opportunity to train multiple times or were exposed to the instructions too many times. However, it could be argued that a more valid comparison would be made if participants were able to train for the same amount of time and could complete the model or watch the video several times until they felt they were trained well-enough.

Previous studies have largely focused on using VR as a training tool for gaining factual knowledge, with some promising results (e.g., Logishetty et al., 2019). However, only a small number of studies have used objective performance indexes to evaluate the trainings, and they commonly rely on subjective reports on questionnaires. It is therefore necessary to test skills and knowledge learning using objective indexes, as it was attempted in the present study.

Furthermore, for the many variables at play, our results may only be valid for a building task, and thus difficult to generalize to other types of training. It is also arguable that the task trained in the present study may be not comparable to more complex tasks, like the ones needed to train industrial procedures. The trainings for many hazardous industries involve complicated processes that integrate various set of skills, and they do not only involve procedural memory.

The inclusion of both procedural skills and specific knowledge about the procedures in a VR simulation has been previously attempted, with promising results. Dubovi et al. (2017) studied the effect of VR-based teaching method for the higher education of professionals requiring a combination of theoretical knowledge and practical skills (nursing and medical skills). Their results revealed significantly better conceptual and procedural knowledge learning gains from the VR simulation compared to traditional lecture-based training.

Another factor that could influence the learning outcome through VR, which the present study did not take into account, is experience with VR. With immersive VR technology becoming more common for personal and entertainment use, several users might have experience with VR. On the other hand, VR might still be a totally new experience for some people, and those individuals might have a harder time adapting to the VR environment and optimally handling the controllers. Since it is probable that there was a difference in VR capability among the participants, this factor may have affected the result for the VR session in an uncontrollable way.

Results from the present study are difficult to generalize in the wider context of VR. For example, the task that participants were asked to complete did not involve physical locomotion. Many users report of negative side effects relating to HMDs the context of virtual locomotion (see e.g., Saredakis et al., 2020).

Finally, one of the conditions this study took for granted was that participants all had some degree of previous experience with building blocks. Future studies could control the level of pre-existing skills of the trainee, and eventually exclude those that have either too much of too little experience in the training task.

## Conclusion

The present investigation aimed to evaluate whether immersive VR technology could increase skill learning more than a less immersive instructional 2D video. In the present investigation, we failed to find a statistically significant increase in performance in users training using HMD-mediated VR compared to users trained using a traditional instruction video presented on a 2D screen. However, we acknowledge that such lack of difference in the two groups may be due to limitations in the experimental design. Furthermore, male and female participants did not differ in any of the objective performance metrics and did not experience the VR environment differently (with regard to sense of presence and level of SS), based on subjective reports. The reported sense of presence experienced during the VR training was highly correlated with all the performance metrics. Such results suggest that developing VR environments that promote a high sense of presence in the user may improve the user’s performances after VR-based training. Future studies may attempt to use psychophysiological measures to better understand the relationship immersive environments, training, and sense of presence.

## Data Availability Statement

The datasets presented in this study can be found in online repositories. The names of the repository/repositories and accession number(s) can be found below: DOI: 10.5281/zenodo.3740071.

## Ethics Statement

This study was reviewed and approved by Norwegian Centre for Research Data (NSD). The patients/participants provided their written informed consent to participate in this study.

## Author Contributions

SG was responsible for the study design, laboratory work supervision, data analysis, and writing the present manuscript. KL provided funding for the present research, gave feedback on all the phases of the project, and reviewed and commented on the present manuscript. MS gave feedbacks on all the phases of the project and reviewed and commented on the present manuscript. All the authors contributed to develop the idea of the study and supervised the development of the VR software that was used for the study. All authors contributed to the article and approved the submitted version.

### Conflict of Interest

The authors declare that the research was conducted in the absence of any commercial or financial relationships that could be construed as a potential conflict of interest.
